# Rapid growth of new atmospheric particles by nitric acid and ammonia condensation

**DOI:** 10.1038/s41586-020-2270-4

**Published:** 2020-05-13

**Authors:** Mingyi Wang, Weimeng Kong, Ruby Marten, Xu-Cheng He, Dexian Chen, Joschka Pfeifer, Arto Heitto, Jenni Kontkanen, Lubna Dada, Andreas Kürten, Taina Yli-Juuti, Hanna E. Manninen, Stavros Amanatidis, António Amorim, Rima Baalbaki, Andrea Baccarini, David M. Bell, Barbara Bertozzi, Steffen Bräkling, Sophia Brilke, Lucía Caudillo Murillo, Randall Chiu, Biwu Chu, Louis-Philippe De Menezes, Jonathan Duplissy, Henning Finkenzeller, Loic Gonzalez Carracedo, Manuel Granzin, Roberto Guida, Armin Hansel, Victoria Hofbauer, Jordan Krechmer, Katrianne Lehtipalo, Houssni Lamkaddam, Markus Lampimäki, Chuan Ping Lee, Vladimir Makhmutov, Guillaume Marie, Serge Mathot, Roy L. Mauldin, Bernhard Mentler, Tatjana Müller, Antti Onnela, Eva Partoll, Tuukka Petäjä, Maxim Philippov, Veronika Pospisilova, Ananth Ranjithkumar, Matti Rissanen, Birte Rörup, Wiebke Scholz, Jiali Shen, Mario Simon, Mikko Sipilä, Gerhard Steiner, Dominik Stolzenburg, Yee Jun Tham, António Tomé, Andrea C. Wagner, Dongyu S. Wang, Yonghong Wang, Stefan K. Weber, Paul M. Winkler, Peter J. Wlasits, Yusheng Wu, Mao Xiao, Qing Ye, Marcel Zauner-Wieczorek, Xueqin Zhou, Rainer Volkamer, Ilona Riipinen, Josef Dommen, Joachim Curtius, Urs Baltensperger, Markku Kulmala, Douglas R. Worsnop, Jasper Kirkby, John H. Seinfeld, Imad El-Haddad, Richard C. Flagan, Neil M. Donahue

**Affiliations:** 1 Center for Atmospheric Particle Studies, Carnegie Mellon University, Pittsburgh, PA USA; 2 Department of Chemistry, Carnegie Mellon University, Pittsburgh, PA USA; 3 Division of Chemistry and Chemical Engineering, California Institute of Technology, Pasadena, CA USA; 4 Laboratory of Atmospheric Chemistry, Paul Scherrer Institute, Villigen, Switzerland; 5 Institute for Atmospheric and Earth System Research (INAR), University of Helsinki, Helsinki, Finland; 6 Department of Chemical Engineering, Carnegie Mellon University, Pittsburgh, PA USA; 7 CERN, the European Organization for Nuclear Research, Geneva, Switzerland; 8 Department of Applied Physics, University of Eastern Finland, Kuopio, Finland; 9 Institute for Atmospheric and Environmental Sciences, Goethe University Frankfurt, Frankfurt am Main, Germany; 10 CENTRA and Faculdade de Ciências da Universidade de Lisboa, Campo Grande, Lisbon Portugal; 11 Institute of Meteorology and Climate Research, Karlsruhe Institute of Technology, Karlsruhe, Germany; 12 Tofwerk, Thun, Switzerland; 13 Faculty of Physics, University of Vienna, Vienna, Austria; 14 Department of Chemistry and CIRES, University of Colorado at Boulder, Boulder, CO USA; 15 Helsinki Institute of Physics, University of Helsinki, Helsinki, Finland; 16 Institute for Ion Physics and Applied Physics, University of Innsbruck, Innsbruck, Austria; 17 Ionicon Analytik, Innsbruck, Austria; 18 Aerodyne Research, Billerica, MA USA; 19 Finnish Meteorological Institute, Helsinki, Finland; 20 P.N. Lebedev Physical Institute of the Russian Academy of Sciences, Moscow, Russia; 21 Department of Atmospheric and Oceanic Sciences, University of Colorado at Boulder, Boulder, CO USA; 22 School of Earth and Environment, University of Leeds, Leeds, UK; 23 Aerosol Physics Laboratory, Physics Unit, Faculty of Engineering and Natural Sciences, Tampere University, Tampere, Finland; 24 Grimm Aerosol Technik Ainring, Ainring, Germany; 25 Institute Infante Dom Luíz, University of Beira Interior, Covilhã, Portugal; 26 Department of Engineering and Public Policy, Carnegie Mellon University, Pittsburgh, PA USA; 27 Department of Applied Environmental Science, University of Stockholm, Stockholm, Sweden; 28 Joint International Research Laboratory of Atmospheric and Earth System Sciences, Nanjing University, Nanjing, China; 29 Aerosol and Haze Laboratory, Beijing Advanced Innovation Center for Soft Matter Science and Engineering, Beijing University of Chemical Technology, Beijing, China

**Keywords:** Atmospheric science, Climate change

## Abstract

A list of authors and their affiliations appears at the end of the paper New-particle formation is a major contributor to urban smog^1,2^, but how it occurs in cities is often puzzling^3^. If the growth rates of urban particles are similar to those found in cleaner environments (1–10 nanometres per hour), then existing understanding suggests that new urban particles should be rapidly scavenged by the high concentration of pre-existing particles. Here we show, through experiments performed under atmospheric conditions in the CLOUD chamber at CERN, that below about +5 degrees Celsius, nitric acid and ammonia vapours can condense onto freshly nucleated particles as small as a few nanometres in diameter. Moreover, when it is cold enough (below −15 degrees Celsius), nitric acid and ammonia can nucleate directly through an acid–base stabilization mechanism to form ammonium nitrate particles. Given that these vapours are often one thousand times more abundant than sulfuric acid, the resulting particle growth rates can be extremely high, reaching well above 100 nanometres per hour. However, these high growth rates require the gas-particle ammonium nitrate system to be out of equilibrium in order to sustain gas-phase supersaturations. In view of the strong temperature dependence that we measure for the gas-phase supersaturations, we expect such transient conditions to occur in inhomogeneous urban settings, especially in wintertime, driven by vertical mixing and by strong local sources such as traffic. Even though rapid growth from nitric acid and ammonia condensation may last for only a few minutes, it is nonetheless fast enough to shepherd freshly nucleated particles through the smallest size range where they are most vulnerable to scavenging loss, thus greatly increasing their survival probability. We also expect nitric acid and ammonia nucleation and rapid growth to be important in the relatively clean and cold upper free troposphere, where ammonia can be convected from the continental boundary layer and nitric acid is abundant from electrical storms^4,5^.

## Abstract (web summary)

Measurements in the CLOUD chamber at CERN show that the rapid condensation of ammonia and nitric acid vapours could be important for the formation and survival of new particles in wintertime urban conditions, contributing to urban smog.

## Main

The formation of new particles may mask up to half of the radiative forcing caused since the industrial revolution by carbon dioxide and other long-lived greenhouse gases^6^. Present-day particle formation is thought to predominantly involve sulfuric acid vapours globally^7–9^. Subsequent particle growth is richer, often involving organic molecules^10^. Often growth is the limiting step for the survival of particles from freshly nucleated clusters to diameters of 50 or 100 nm, where they become large enough to directly scatter light and also to seed cloud formation^11,12^.

**Extended Data Fig. 1:**
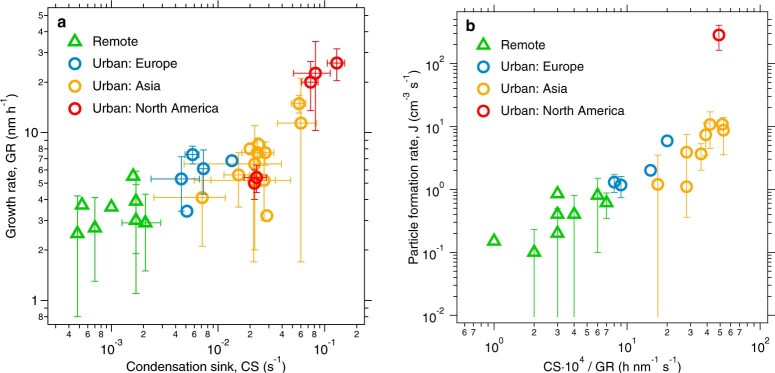
New-particle-formation events observed in various remote and urban environments (see Extended Data Table 3 for a complete set of references). **a**, Growth rates (GR) versus condensation sinks (CS), showing that both are higher in polluted urban environments than in other environments. **b**, Particle-formation rates (*J*) versus a measure of particle loss via coagulation (CS × 10^4^/GR, similar to the the McMurry *L* parameter), showing high new-particle-formation rates in urban conditions where the condensation sinks were so high compared to the growth rate that survival of nucleated particles should be very low. *J* and GR were calculated over the size range from a few nanometres to over 20 nm, except for *J* at Shanghai^59^ and Tecamac^60^, which were calculated from 3 nm to 6 nm. The bars indicate 1*σ* total errors. Source Data

New-particle formation in megacities is especially important^2^, in part because air pollution in megacities constitutes a public health crisis^13^, but also because the regional climate forcing associated with megacity urban haze can be large^14^. However, new-particle formation in highly polluted megacities is often perplexing, because the apparent particle growth rates are only modestly faster (by a factor of roughly three) than growth rates in remote areas, whereas the vapour condensation sink (to background particles) is up to two orders of magnitude larger (Extended Data Fig. 1). This implies a very low survival probability in the ‘valley of death’, where particles with diameters (*d*_p_) of 10 nm or less have high Brownian diffusivities and will be lost by coagulational scavenging unless they grow rapidly^7,15^.

Ammonium nitrate has long been recognized as an important yet semivolatile constituent of atmospheric aerosols^16^. Especially in winter and in agricultural areas, particulate nitrate can be a substantial air-quality problem^17^. However, the partitioning of nitric acid and ammonia vapours with particulate ammonium nitrate is thought to rapidly reach an equilibrium, often favouring the gas phase when it is warm.

Because ammonium nitrate is semivolatile, nitric acid has not been thought to play an important role in new-particle formation and growth, where very low vapour pressures are required for constituents to be important. Such constituents would include sulfuric acid^18^ but also very low vapour pressure organics^19,20^ and iodine oxides^21^. However, it is saturation ratio and not vapour pressure per se that determines the thermodynamic driving force for condensation, and nitric acid can be three or four orders of magnitude more abundant than sulfuric acid in urban environments. Thus, even a small fractional supersaturation of nitric acid and ammonia vapours with respect to ammonium nitrate has the potential to drive very rapid particle growth, carrying very small, freshly nucleated particles through the valley of death in a few minutes. These rapid growth events can exceed 100 nm h^−1^ under urban conditions—an order of magnitude higher than previous observations—and the growth will continue until the vapours are exhausted and conditions return to equilibrium. Such transients will be difficult to identify in inhomogeneous urban environments, yet have the potential to explain the puzzling observations of new-particle formation in highly polluted megacities.

## Nucleation measurements in CLOUD at CERN

**Fig. 1:**
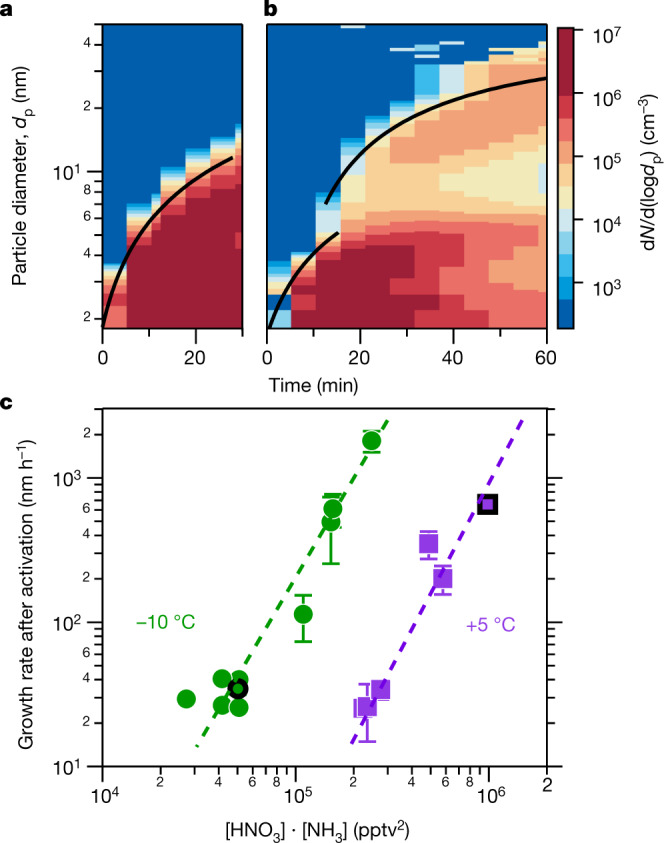
Rapid growth events observed in the CERN CLOUD chamber. **a**, Particle nucleation and growth (particle growth rate, d*d*_*p*_/d*t*) at −10 °C from a mixture of 0.44 pptv sulfuric acid and 1,915 pptv ammonia at 60% relative humidity. Particles form and grow to roughly 10 nm in 30 min. The black curve shows the linear fit to the 50% appearance times. **b**, Particle formation and growth under identical conditions to those in **a**, but with the addition of 24 pptv of nitric acid vapour formed via NO_2_ oxidation. Once particles reach roughly 5 nm, they experience rapid growth to much larger sizes, reaching more than 30 nm in 45 min. **c**, Observed growth rates after activation versus the product of measured nitric acid and ammonia levels at +5 °C and −10 °C. The point corresponding to the rapid growth regime for *d*_p_ > 6 nm in **b** is a black-outlined green circle, and the point corresponding to Fig. 2 is a black-outlined purple square. Growth rates at a given vapour product are substantially faster at −10 °C than at +5 °C, consistent with semivolatile condensation that is rate limited by ammonium nitrate formation. Error bars are 95% confidence limits on the fitting coefficients used to determine growth rates. The overall systematic scale uncertainties of ±10% on the NH_3_ mixing ratio and ±25% on the HNO_3_ mixing ratio are not shown. Source Data

Here we report experiments performed with mixtures of nitric acid, sulfuric acid and ammonia vapours under atmospheric conditions in the CERN CLOUD chamber (Cosmics Leaving OUtdoor Droplets^22^; see Methods for experimental details) from 21 September to 7 December 2018 (CLOUD 13). We varied the temperature from +20 °C to −10 °C, in one case cooling progressively from −15 °C to −25 °C. We adjusted levels of sulfuric acid (H_2_SO_4_), ammonia (NH_3_) and nitric acid (HNO_3_), as well as aromatic precursors, to span the ranges typical of polluted megacities. In Fig. 1 we show two representative events at −10 °C. For Fig. 1a we oxidized SO_2_ with OH to form H_2_SO_4_ in the presence of 1,915 parts per trillion volume (pptv) ammonia. The resulting ‘banana’ is typical of such experiments and of ambient observations under relatively clean conditions, with a single nucleation mode that appears shortly after the onset of nucleation and grows at roughly 20 nm h^−1^. In Fig. 1b we repeated this experiment but also with 5.8 parts per billion volume (ppbv) NO_2_, which was oxidized by OH to produce 24 pptv of HNO_3_ vapour. The resulting size distribution initially resembles the first case, but when the particles reach about 5 nm, their growth rate accelerates to roughly 45 nm h^−1^. This activation is reminiscent of cloud-droplet activation and thus suggestive of ‘nano-Köhler’ behaviour and the Kelvin curvature effect^23^.

We repeated these experiments over a range of conditions, either forming HNO_3_ from NO_2_ oxidation or injecting it directly into the CLOUD chamber from an ultrapure evaporation source. We observed this activation and rapid growth behaviour consistently. In Fig. 1c we show the resulting rapid growth rates after activation at −10 °C (green) and +5 °C (purple), plotted against the product of the measured gas-phase HNO_3_ and NH_3_ mixing ratios. Growth rates are based on the 50% appearance time—the time at which particle number concentrations in each size bin of the rapid growth regime reach 50% of their maximum. Both a strong correlation and a clear temperature dependence are evident; when it is colder, the particles grow at the same rate for a much lower product of vapour concentrations. This is consistent with semivolatile uptake of both species, rate limited by the formation of ammonium nitrate.

**Fig. 2:**
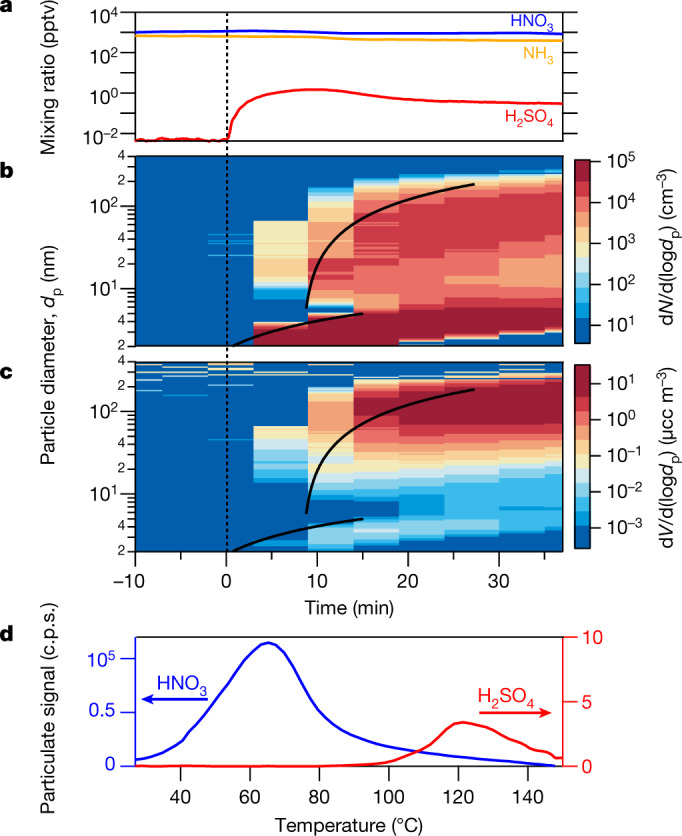
Chemical composition during a rapid growth event at +5 °C and 60% relative humidity. This growth event is indicated in Fig. 1c with a black-outlined purple square. **a**, Gas-phase nitric acid (NO_3_), ammonia (NH_3_) and sulfuric acid (H_2_SO_4_) mixing ratios versus time in an event initiated by SO_2_ oxidation, with constant nitric acid and ammonia. **b**, Particle diameters and number distributions versus time, showing a clean chamber (to the left of the vertical dotted line), then nucleation after sulfuric acid formation and rapid growth once particles reach 2.3 nm. Black curves are linear fits to the 50% appearance times. **c**, Particle volume distributions from the same data, showing that 200-nm particles dominate the mass after 15 min. 1 μcc = 1 cm^−6^. **d**, FIGAERO thermogram from a 30-min filter sample after rapid growth (c.p.s., counts per second). The particle composition is dominated by nitrate with a core of sulfate, consistent with rapid growth by ammonium nitrate condensation on an ammonium sulfate (or bisulfate) core (note the different *y*-axis scales; the instrument is not sensitive to ammonia). A thermogram from just before the formation event shows no signal from either nitrate or sulfate, indicating that vapour adsorption did not interfere with the analysis. Source Data

To confirm this, we measured the composition of the particles using a filter inlet for gases and aerosols (FIGAERO) iodide (I^−^) chemical ionization mass spectrometer (CIMS), along with the gas-phase vapour concentrations via several CIMS methods. In Fig. 2 we show another rapid growth event, this one at +5 °C (indicated in Fig. 1c with a black outlined purple square). We started with an almost perfectly clean chamber and only vapours present (SO_2_, HNO_3_ and NH_3_) at constant levels (Fig. 2a). Here we injected the HNO_3_ without photochemical production so we could independently control HNO_3_ and sulfuric acid. The FIGAERO showed no measurable signal in the absence of particles, indicating negligible crosstalk from vapours. We then turned on ultraviolet lights in order to form OH radicals and to initiate SO_2_ oxidation to H_2_SO_4_. Fig. 2b shows the resulting number distribution; as in Fig. 1b, particles appear, grow slowly, and then activate and grow at 700 nm h^−1^. We again show the 50% appearance time of both modes. In Fig. 2c we show the associated volume distribution. Within 15 min of the onset of particle formation, the volume is dominated by the upper mode near 200 nm. Finally, in Fig. 2d we show a FIGAERO thermogram (signal versus desorption temperature) for particles collected between 10 min and 40 min after the onset of photochemistry. Their composition is dominated by nitrate, with a much smaller but notable sulfate contribution; the semivolatile nitrate desorbs at a much lower temperature than the sulfate. The I^−^ chemical ionization is not sensitive to NH_3_, but both nitrate and sulfate exist presumably as ammonium salts in the particles.

**Fig. 3:**
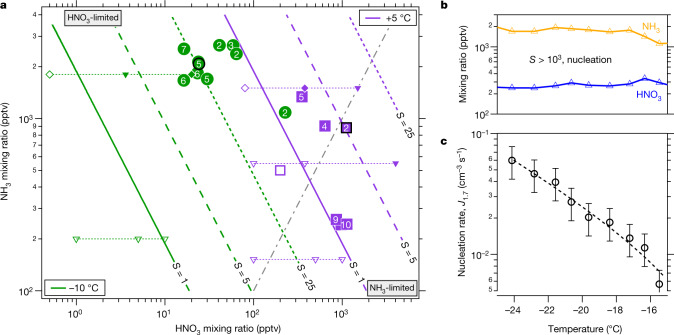
Phase space for rapid growth and nucleation. **a**, Ammonium nitrate saturation ratios versus gas-phase nitric acid and ammonia mixing ratios at 60% relative humidity. The coloured lines (slope = −1) represent *S* = 1 (bold), *S* = 5 (dashed) and *S* = 25 (dotted), at −10 °C (green) and +5 °C (purple). The slope = +1 dot-dashed grey line indicates a 1:1 ammonia:nitric-acid stoichiometry; the phase space to the upper left of this line is nitric-acid limited. Observed activation diameters (in nm) for measured nitric-acid–ammonia pairs are plotted as numbers inside solid circles and squares; open symbols show no activation. Activation occurs only for *S* values of more than 1, and the activation diameter decreases as *S* increases. Points from MABNAG simulations are shown with open triangles for no activation and filled triangles for activation; simulations indicated with diamonds are shown in detail in Fig. 4 and Extended Data Fig. 4. Points from runs shown in Figs. 1, 2 are emphasized with a thick black outline. **b**, Mixing ratios for ammonia and nitric acid vapour during a pure ammonium nitrate nucleation scan from −16 °C to −24 °C. **c**, Particle-formation (nucleation) rates (*J*_1.7_) during the nucleation scan, showing a strong inverse relationship with temperature at constant HNO_3_ and NH_3_, with H_2_SO_4_ concentrations of less than 0.002 pptv and relative humidity starting at 60% and ending at 40%. The bars indicate 30% estimated total errors on the nucleation rates, although the overall systematic scale uncertainties of ±10% on the NH_3_ mixing ratio and ±25% on the HNO_3_ mixing ratio are not shown. Source Data

**Extended Data Fig. 2:**
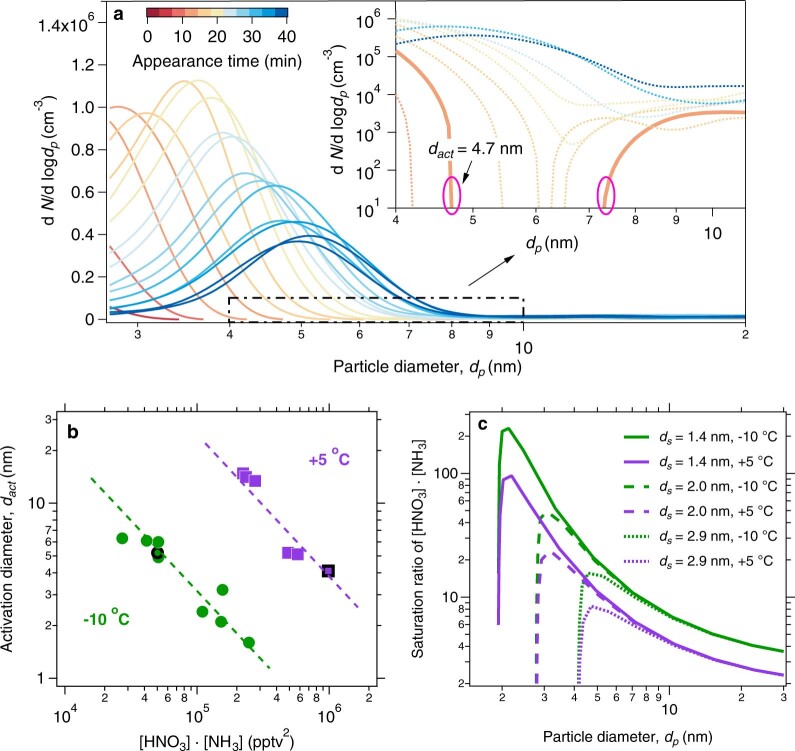
Activation diameter of newly formed particles. **a**, Determination of the activation diameter, *d*_act_, from a rapid growth event at +5 °C, in the presence of nitric acid, ammonia and sulfuric acid. The solid orange trace in the insert indicates the first size distribution curve that exhibited a clear bimodal distribution, which appeared roughly 7 min after nucleation. We define the activation diameter as the largest observed size of the smaller mode. In this case, *d*_act_ = 4.7 nm, which agrees well with the value obtained from MABNAG simulations (roughly 4 nm) under the same conditions as in Fig. 4. **b**, Activation diameter versus vapour product. Measured activation diameters at a given temperature correlate inversely with the product of nitric acid and ammonia vapours, in a log-log space. An amount of vapour product that is approximately one order of magnitude higher is required for the same *d*_act_ at +5 °C than at −10 °C, because of the higher vapour pressure (faster dissociation) of ammonium nitrate when it is warmer. **c**, Equilibrium particle diameter (*d*_p_) at different saturation ratios of ammonium nitrate, calculated according to nano-Köhler theory. Purple curves are for +5 °C and green curves are for −10 °C (as throughout this work). The line type shows the diameter of the seed particle (*d*_s_). The maximum of each curve corresponds to the activation diameter (*d*_act_). A higher supersaturation is required for activation at lower temperature. Source Data

In addition to the correlation of activated particle growth rates with the product of HNO_3_ and NH_3_ at a given temperature, the observed activation diameter (*d*_act_) shows a strong dependence on this product. The activation diameter is evident as a clear kink in the 50% appearance curve, as well as a notable absence of particles in the slower-growth mode above *d*_act_. In Extended Data Fig. 2a we show an example of how we determine *d*_act_, using the emergence of a bimodal size distribution as the defining feature. In Fig. 3a we plot the observed activation diameter at each temperature in a phase space, with [HNO_3_] on the log*x* axis and [NH_3_] on the log*y* axis (both in pptv). The number within each symbol is the observed activation diameter for that experiment. We show the saturation ratio (*S*) of ammonium nitrate at each temperature via a series of diagonal lines in this log–log space (slope = −1); specifically, we show *S* = 1, 5 and 25, emphasizing *S* = 1 as a thick solid line. We also indicate 1:1 [HNO_3_]:[NH_3_] with a dashed grey line (slope +1); points to the upper left (most of the values) are ‘nitric acid limited’, with more ammonia than nitric acid. All of these concentrations are well within the ranges typically observed in wintertime megacity conditions^24^.

For both +5 °C and −10 °C, we consistently observe a relationship between *S* and *d*_act_ (we never achieved saturation at +20 °C and did not observe rapid growth). We observe no activation for *S* values of less than 1, and activation for *S* values greater than 1, with log*d*_act_ being inversely proportional to log([HNO_3_]⋅[NH_3_]) at each temperature (Extended Data Fig. 2b). Notably, *d*_act_ can be well under 10 nm and as low as 1.6 nm. This suggests that nitric acid and ammonia (ammonium nitrate) condensation may play a role in new-particle formation and growth within the valley of death, where very small particles are most vulnerable to loss by coagulation^8^.

**Extended Data Fig. 3:**
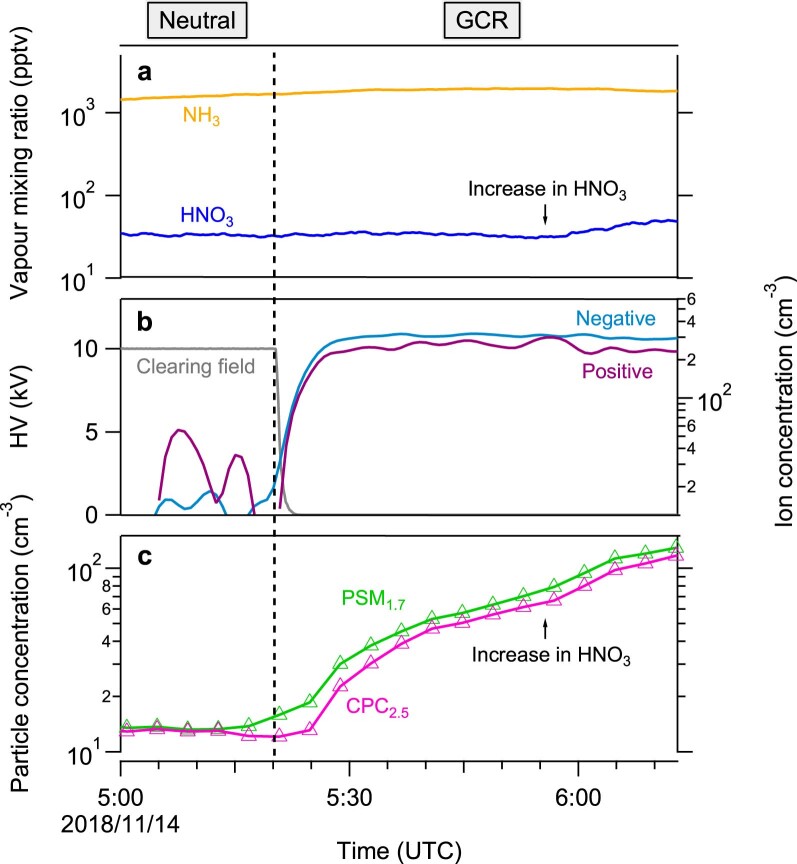
A typical measurement sequence. Nucleation was carried out purely from nitric acid and ammonia, with no sulfuric acid (measured to less than 5 × 10^4^ cm^−3^ or 2 × 10^−3^ pptv), as a function of coordinated universal time (UTC), at 60% relative humidity and −25 °C. **a**, Gas-phase ammonia and nitric acid mixing ratios. The run started with injection of the nitric acid and ammonia flow into the chamber to reach chosen steady-state values near 30 pptv and 1,500 pptv, respectively. The nitric acid flow was increased at 5:53 on 14 November 2018 to prove consistency. **b**, Clearing-field voltage and ion concentrations. Primary ions were formed from galactic cosmic rays (GCR). The clearing-field high voltage (HV) was used to sweep out small ions at the beginning of the run, and turned off at 05:21 on 14 November 2018 to allow the ion concentration to build up to a steady state between GCR production and wall deposition. **c**, Particle concentrations at two cut-off sizes (1.7 nm and 2.5 nm). Particles formed slowly in the chamber under ‘neutral’ conditions with the HV clearing field on and thus without ions present. The presence of ions (‘GCR’ condition) caused a sharp increase in the particle-number concentration by about one order of magnitude, with a slower approach to steady state because of the longer wall-deposition time constant for the larger particles. Particle numbers rose again with rising nitric acid. Source Data

We also performed experiments with only nitric acid, ammonia and water vapour added to the chamber (sulfuric acid contamination was measured to be less than 2 × 10^−3^ pptv). For temperatures of less than −15 °C and *S* values of more than 10^3^, we observed nucleation and growth of pure ammonium nitrate particles (Fig. 3c). We progressively cooled the chamber to −24 °C, while holding the vapours at a constant level (Fig. 3b). The particle-formation rate (*J*_1.7_) rose steadily from 0.006 cm^−3^ s^−1^ to 0.06 cm^−3^ s^−1^ at −24 °C. In Extended Data Fig. 3 we show a pure ammonium nitrate nucleation experiment performed at −25 °C under vapour conditions reported for the tropical upper troposphere^4^ (30–50 pptv nitric acid and 1.8 ppbv ammonia), showing that this mechanism can produce several 100 cm^−3^ particles per hour.

**Fig. 4:**
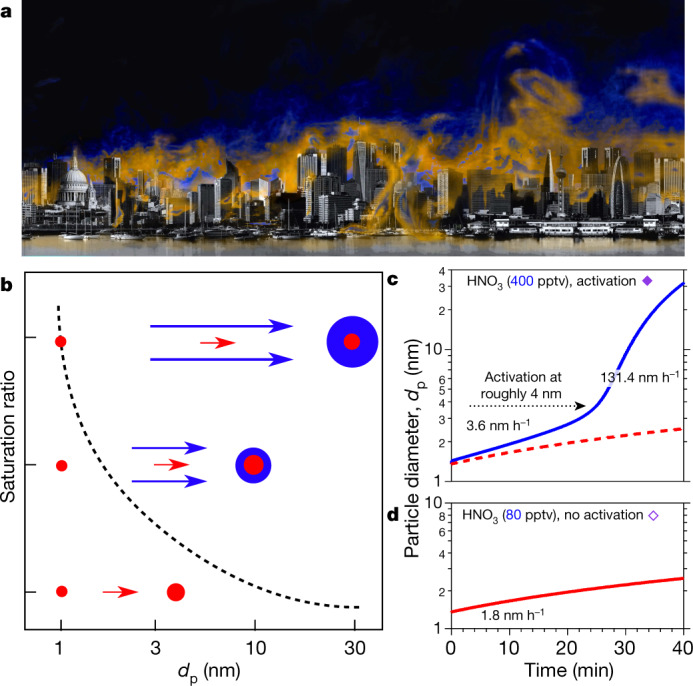
Conditions for rapid growth. Persistent supersaturations of ammonia and nitric acid with respect to ammonium nitrate will be sustained by inhomogeneity in urban conditions with high source strength. This will be sufficient to accelerate particle growth in the range 1–10 nm, where survival is threatened by the high coagulation sink of pre-existing particles from pollution. **a**, Conceptual image of urban conditions, where inhomogeneities in the concentrations of ammonia and nitric acid vapour and in temperatures are caused by non-uniform sources and large-scale eddies. **b**, Particles nucleate and grow slowly as (base-stabilized) sulfate (red). The activation size (shown with *d*_p_ on the *x*-axis) correlates inversely with the ammonium nitrate saturation ratio (shown qualitatively on the *y*-axis), as indicated by the dashed curve. Available concentrations of gas-phase nitric acid can exceed those of sulfuric acid by a factor of 1,000, so modest supersaturation drives rapid growth (blue) above an activation diameter determined by particle curvature (the Kelvin term). **c**, **d**, Monodisperse thermodynamic growth calculations (from MABNAG simulations) for high (**c**) and low (**d**) saturation ratios of ammonium nitrate, corresponding to **b** and to the closed and open diamonds towards the upper right in Fig. 3a. For a saturation ratio near 4, activation is predicted to occur near 4 nm, consistent with our observations. Source Data

**Extended Data Fig. 4:**
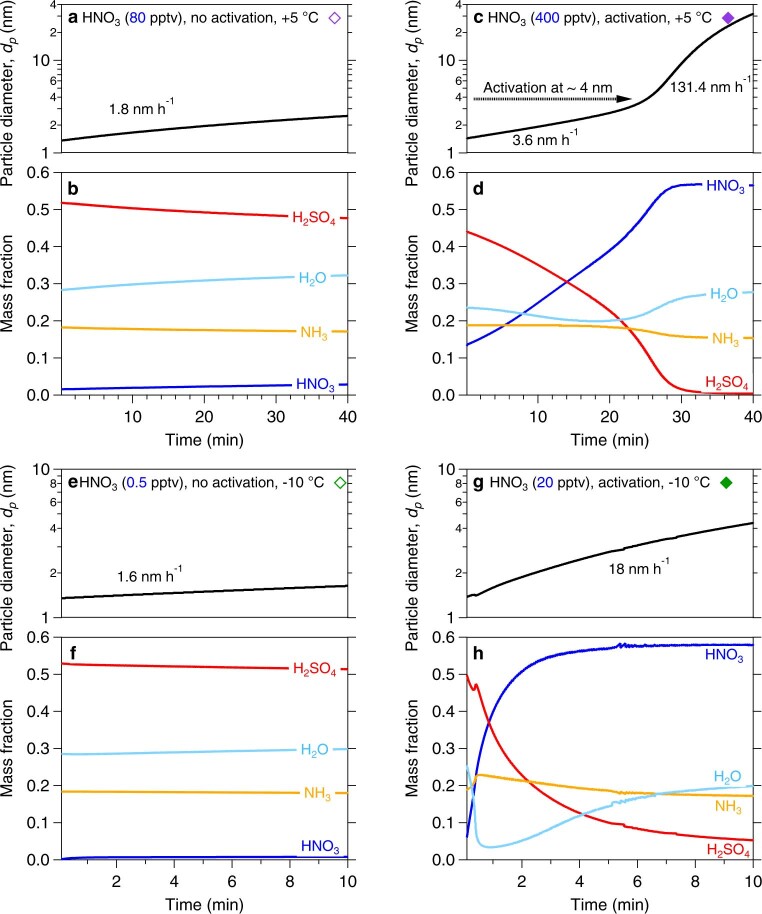
Comparison of growth rates and chemical composition in four simulations at +5 °C and −10 °C with the thermodynamic model MABNAG. The simulation points are shown in Fig. 3a (filed diamonds, with activation; open diamonds, without activation). **a**, **c**, **e**, **g**, Temporal evolution of the particle diameter. **b**, **d**, **f**, **h**, Temporal evolution of the particle-phase chemical composition. The left-hand column (**a**, **b**, **e**, **f**) shows simulations without activation. The right-hand column (**c**, **d**, **g**, **h**) shows simulations with activation. We set the HNO_3_ mixing ratios at 80 pptv and 400 pptv with 1,500 pptv NH_3_ at +5 °C, and set the HNO_3_ mixing ratios at 20 pptv and 0.5 pptv with 1,500 pptv NH_3_ at −10 °C, to simulate unsaturated (**a**, **b**, **e**, **f**) and supersaturated (**c**, **d**, **g**, **h**) conditions, respectively. All other conditions were held constant for the simulations, with the [H_2_SO_4_] at 2 × 10^7^ cm^−3^ and relative humidity at 60%. Activation corresponds to a rapid increase in the nitric acid (nitrate) mass fraction; the simulations for activation conditions suggest that water activity may be an interesting variable influencing activation behaviour. The activated model results (**c**, **d**, **g**, **h**) confirm that supersaturated nitric acid and ammonia lead to rapid growth of nanoparticles. The simulated activation diameter at +5 °C is roughly 4 nm, similar to that from the chamber experiment (4.7 nm, Fig. 3a); at −10 °C the simulated activation diameter is less than 2 nm, smaller than observed. Source Data

Our experiments show that semivolatile ammonium nitrate can condense on tiny nanoparticles, consistent with nano-Köhler theory^23^. To confirm this we conducted a series of simulations using the monodisperse thermodynamic model MABNAG (model for acid-base chemistry in nanoparticle growth)^25^, which treats known thermodynamics, including curvature (Kelvin) effects for a single evolving particle size. We show the points of the MABNAG simulations as triangles in Fig. 3a. MABNAG consistently and quantitatively confirms our experimental findings: there is little ammonium nitrate formation at *S* values of less than 1.0, as expected; and activation behaviour with ammonium nitrate condensation ultimately dominating the particle composition occurs at progressively smaller *d*_act_ values as *S* rises well above 1.0. The calculated and observed *d*_act_ values are broadly consistent. In Fig. 4 we show two representative MABNAG growth simulations for the two points indicated with open and filled diamonds in Fig. 3a; the simulations show no ammonium nitrate formation when conditions are undersaturated, but substantial formation when conditions are saturated, with activation behaviour near the observed *d*_act_ = 4.7 nm. We show the calculated composition as well as diameter versus time for these and other cases in Extended Data Fig. 4.

We also conducted nano-Köhler simulations^23^, shown in Extended Data Fig. 2c, which confirm the activation of ammonium nitrate condensation at diameters less than 4 nm, depending on the size of an assumed ammonium sulfate core. For a given core size the critical supersaturation required for activation at −10 °C is a factor of two to three higher than at +5 °C, consistent with the observed behaviour shown in Fig. 3a. While particles of 1–2 nm contain only a handful of acid and base molecules, the MABNAG and nano-Köhler simulations based on bulk thermodynamics—with only a Kelvin term to represent the unique behaviour of the nanoparticles—capture the activation and growth behaviours we observe.

## Atmospheric implications

Our findings suggest that the condensation of nitric acid and ammonia onto nanoparticles to form ammonium nitrate (or, by extension, aminium nitrates in the presence of amines) may be important in the atmosphere. This process may contribute to urban new-particle formation during wintertime via rapid growth. It may also play a role in free-tropospheric particle formation, where sufficient vapours may exist to allow nucleation and growth of pure ammonium nitrate particles. We observe these behaviours in CLOUD for vapour concentrations well within those typical of the atmosphere.

Rapid growth may contribute to the often puzzling survival of newly formed particles in megacities, where particles form at rates consistent with sulfuric-acid–base nucleation and appear to grow at typical rates (roughly 10 nm h^−1^) in the presence of extremely high condensation sinks that seemingly should scavenge all of the tiny nucleated particles. As shown in Extended Data Fig. 1, the ratio of 10^4^ × condensation sink (CS; in units of s^−1^) to growth rate (GR; in nm h^−1^) during nucleation events in Asian megacities typically ranges between 20 and 50, where the survival probability of particles with sizes of between 1.5 nm and 3 nm should drop precipitously^3^. However, the observed growth rates are based on appearance times in measured ambient size distributions—just as in Figs. 1, 2—and thus reflect a spatial and temporal average of air masses passing over a sampling site during the course of a day. Rapid growth rates can reduce CS:GR by a factor of ten or more, effectively displacing urban ratios into a range characteristic of remote regions (Extended Data Fig. 1b). The empirically derived nucleation rates in Extended Data Fig. 1b correlate positively with high CS:GR, consistent with high production rates of condensable vapours; however, the complicated microphysics of particles smaller than 10 nm make a simple determination of the growth rate difficult. Urban conditions are however far less homogeneous than those of CLOUD, or even of remote boreal forests such as Hyytiälä. Because survival probability depends exponentially on CS:GR (refs. ^3,7^), but spatial (and temporal) averaging as well as ambient mixing are linear, real urban conditions may contain pockets conducive to transient rapid growth and thus unusually high survival probability that are blurred in the (averaged) observations.

The key here is that nitric acid vapour and ammonia are often at least one thousand times more abundant than sulfuric acid vapour. Thus, although they tend towards equilibrium with ammonium nitrate in the particle phase, even a modest perturbation above saturation can unleash a tremendous thermodynamic driving force for condensational growth, nominally up to one thousand times faster than growth by sulfuric acid condensation. This may be brief, but because of the disparity in concentrations, even a small deviation in saturation ratio above 1.0 may drive rapid growth for a short period at several nanometres per minute, as opposed to several nanometres per hour. The particles will not experience rapid growth for long, but they may grow fast enough to escape the valley of death.

We illustrate rapid growth in Fig. 4. Under most urban conditions, nucleation and early growth up to the activation size are likely to be controlled by sulfuric acid and a base (ammonia or an amine), shown by the red ‘cores’ in Fig. 4b. During the day (even in wintertime)—when NO_2_ is oxidized by OH in the gas phase to produce nitric acid at rates of up to 3 ppbv h^−1^, and ammonia from traffic, other combustion emissions and agriculture can reach 8 ppbv (ref. ^24^)—nitric acid and ammonia will not equilibrate, but rather will approach a modest steady-state supersaturation that drives ammonium nitrate formation to balance the production and emissions. However, this steady state will only be reached after several e-folding time periods set by the particle condensation sink. Typically, new-particle formation occurs at the lower end of the condensation-sink distribution (even under urban conditions)^2,7^, so this timescale will be several minutes, or a length scale of hundreds of metres in the horizontal and tens of metres in the vertical. There are ample sources of inhomogeneity on this timescale, including inhomogeneous sources such as traffic on major roadways and vertical mixing (with an adiabatic lapse rate of −9 °C km^−1^)^24^. Further, large eddy simulations of a megacity (Hong Kong) confirm widespread eddies with spatial scales of tens to hundreds of metres and velocity perturbations of the order 1 m s^−1^ (ref. ^26^). This is consistent with the sustained inhomogeneity required for the rapid growth we demonstrate here, shown conceptually in Fig. 4a. It is thus likely that dense urban conditions will typically include persistent inhomogeneities that maintain supersaturation of nitric acid and ammonia with sufficient magnitude to drive rapid growth, as indicated by the blue ‘shell’ in Fig. 4b. Our thermodynamic models support the phenomenology of Fig. 4b, as shown in Fig. 4c, d, although the composition is likely to be an amorphous mixture of salts (Extended Data Fig. 4). Rapid growth may be sufficient for particles to grow from vulnerable sizes near 2.5 nm to more robust sizes larger than 10 nm. For example, repeated nucleation bursts with very rapid growth were observed in the ammonia- and nitric-acid-rich Cabauw site in the Netherlands during the EUCAARI campaign^27^.

It is common for chemical transport models to use an equilibrium assumption for ammonium nitrate partitioning, because—on the timescale of the coarse spatial grids and long time steps characteristic of large-scale models—the ammonium nitrate aerosol system should equilibrate with respect to the bulk submicrometre-size particles. Further, because rapid growth appears to be rate limited by the formation of ammonium nitrate, the covariance of base and nitric acid sources and concentrations may be essential. Even typical megacity steady-state vapour concentrations fall somewhat above the green points in Fig. 3a (towards larger mixing ratios). For constant production rates, as the temperature falls the ammonium nitrate saturation lines shown in Fig. 3a will sweep from the upper right towards the lower left, moving the system from rough equilibrium for typical urban production and emission rates when it is warmer than about +5 °C, to a sustained supersaturation when it is colder. Just as equilibrium organic condensation and partitioning results in underestimated growth rates from organics in the boreal forest^28^, equilibrium treatments of ammonium nitrate condensation will underestimate the role of nitric acid in nanoparticle growth, especially for inhomogeneous urban environments.

Although the pure ammonium nitrate nucleation rates in Fig. 3c are too slow to compete in urban new-particle formation, this mechanism may provide an important source of new particles in the relatively clean and cold upper free troposphere, where ammonia can be convected from the continental boundary layer^29^ and abundant nitric acid is produced by electrical storms^4^. Theoretical studies have also suggested that nitric acid may serve as a chaperone to facilitate sulfuric-acid–ammonia nucleation^30^. Larger (60–1,000 nm) particles consisting largely of ammonium nitrate, along with more than 1 ppbv of ammonia, have been observed by satellite in the upper troposphere during the Asian monsoon anticyclone^4^, and abundant 3–7-nm particles have been observed in situ in the tropical convective region at low temperature and condensation sink^5^. Although these particles are probably formed via nucleation, the mechanism is not yet known. However, our experiment under similar conditions (Extended Data Fig. 3) shows that it is plausible that pure ammonium nitrate nucleation and/or rapid growth by ammonium nitrate condensation contributes to these particles in the upper troposphere.

Our results indicate that the condensation of nitric acid and ammonia is likely to be an important new mechanism for particle formation and growth in the cold upper free troposphere, as supported by recent observations^4,5^. Furthermore, this process could help to explain how newly formed particles survive scavenging losses in highly polluted urban environments^3^. As worldwide pollution controls continue to reduce SO_2_ emissions sharply, the importance of NO_*x*_ and nitric acid for new-particle formation is likely to increase. In turn, controls on NO_*x*_ and ammonia emissions may become increasingly important, especially for the reduction of urban smog.

## Methods

### The CLOUD facility

We conducted our measurements at the CERN CLOUD facility, a 26.1 m^3^ electropolished stainless-steel chamber that enables new-particle-formation experiments under the full range of tropospheric conditions with scrupulous cleanliness and minimal contamination^22,31^. The CLOUD chamber is mounted in a thermal housing, capable of keeping temperature constant in a range between −65 °C and +100 °C with ±0.1 °C precision^32^, and relative humidity between 0.5% and 101%. Photochemical processes are initiated by homogeneous illumination with a built-in ultraviolet fibre-optic system, including four 200-W Hamamatsu Hg–Xe lamps at wavelengths between 250 nm and 450 nm and a 4-W KrF excimer ultraviolet laser at 248 nm with adjustable power. Ion-induced nucleation under different ionization levels is simulated with a combination of electric fields (±30 kV) and a high-flux beam of 3.6-GeV pions (*π*^+^), which can artificially scavenge or enhance small ions. Uniform spatial mixing is achieved with magnetically coupled stainless-steel fans mounted at the top and bottom of the chamber. The characteristic gas mixing time in the chamber during experiments is a few minutes. The loss rate of condensable vapours and particles onto the chamber walls is comparable to the ambient condensation sink. To avoid contamination, the chamber is periodically cleaned by rinsing the walls with ultrapure water and heating to 100 °C for at least 24 h, ensuring extremely low contaminant levels of sulfuric acid (less than 5 × 10^4^ cm^−3^) and total organics (less than 50 pptv)^19,33^. The CLOUD gas system is also built to the highest technical standards of cleanliness and performance. The dry air supply for the chamber is provided by boil-off oxygen (Messer, 99.999%) and boil-off nitrogen (Messer, 99.999%) mixed at the atmospheric ratio of 79:21. Highly pure water vapour, ozone and other trace gases can be precisely added at the pptv level.

### Typical experimental sequence

To investigate the role of nitric acid in new-particle formation, we performed particle growth experiments at *T* = −10 °C, +5 °C and +20 °C, and (for the most part) at relative humidities of approximately 60%. A typical experiment started with illumination of the chamber at constant ozone (O_3_) to photochemically produce •OH radicals. The subsequent oxidation of premixed SO_2_, NO_2_ and anthropogenic volatile organic compounds (VOCs; that is, toluene or cresol) led to the production of H_2_SO_4_, HNO_3_ and highly oxygenated organic molecules (HOMs), respectively. As a result, nucleation was induced, followed (once the particles reached an activation diameter, *d*_act_) by rapid growth via condensation of nitric acid and ammonia. In some experiments, we also injected nitric acid vapour directly into the CLOUD chamber from an ultrapure source to cover a wide range of conditions. In addition, to prove consistency we also carried out experiments with a biogenic precursor, α-pinene, replacing anthropogenic VOCs, as well as experiments without any organic vapours. For the experiments we focus on here, the HOM concentrations were either zero or small enough to have a minor effect on the experiment. In one case, we cooled the particle-free chamber (with fewer than 1 particle per cm^−3^) continuously from −10 °C to −25 °C, while holding nitric acid and ammonia at a constant level, but with no sulfuric acid (less than 5 × 10^4^ cm^−3^ or 2 × 10^−3^ pptv). We observed new-particle formation purely from nitric acid and ammonia at temperatures of −15 °C and lower. The nucleation rate grew as the temperature dropped. Moreover, as shown in Extended Data Fig. 3, at −25 °C new-particle formation events appeared to be quenched when we swept out primary ions with the electric field, and did not return until the field was turned off to allow primary ion production by galactic cosmic rays to again accumulate (roughly 700 cm^−3^). We list the chamber conditions and key parameters for all the experiments here in Extended Data Table 1.

### Instrumentation

To measure gas-phase nitric acid, we deployed a bromide chemical ionization atmospheric pressure interface time-of-flight (CI-APi-TOF) mass spectrometer^34,35^ equipped with a commercial inlet (Airmodus) to minimize wall contact of the sample^36^. We flowed dibromomethane (CH_2_Br_2_) into the ion-molecule reaction inlet to produce the primary reagent ion Br^−^. During its collision with HNO_3_, Br^−^ reacts either to form a cluster or via a proton transfer from the HNO_3_ to form NO_3_^−^:

$${{\rm{Br}}}^{-}+{{\rm{HNO}}}_{3}\to {{\rm{HNO}}}_{3}\cdot {{\rm{Br}}}^{-}$$

$${{\rm{Br}}}^{-}+{{\rm{HNO}}}_{3}\to {\rm{HBr}}+{{\rm{NO}}}_{3}^{-}$$

To take the variation in the total reagent ions into account, we quantified nitric acid concentrations according to:

$$[{{\rm{HNO}}}_{3}]=\frac{[{{\rm{NO}}}_{3}^{-}]}{[{{\rm{Br}}}^{-}]+[{{\rm{H}}}_{2}{\rm{O}}\cdot {{\rm{Br}}}^{-}]}\times C$$

where *C* (in units of pptv) is a calibration coefficient obtained by measuring HNO_3_/N_2_ mixtures with known nitric acid concentrations. The nitric acid source was a portable permeation tube, kept constantly at 40 °C. An N_2_ flow of 2 litres per minute was introduced into the permeation device to carry out the nitric acid vapour. To determine the permeation rate of nitric acid, we passed the outflow of the permeation tube through an impinger containing deionized water, and analysed the resulting nitric acid solution by spectrophotometry. Line losses during experiments and calibration procedures were calculated separately. We determined the corrected calibration coefficient to be 7,364 pptv.

Gas-phase ammonia was measured by a water cluster CI-APi-TOF mass spectrometer (described elsewhere^37^). The crossflow ion source coupled to a TOF mass spectrometer enables the selective measurement of basic compounds (for example, ammonia) by using positively charged water clusters as primary ions. Owing to the low reaction times (less than 1 ms), the instrument responds rapidly to changing chamber conditions with a detection limit of ammonia of 0.5 pptv.

Gas-phase sulfuric acid and HOMs were routinely measured with a detection limit of approximately 5 × 10^4^ cm^−3^ by two nitrate CI-APi-TOF mass spectrometers. One instrument was equipped with the Airmodus inlet and an X-ray generator as the ion source; the other deployed a home-made inlet and a corona discharge for ion generation^38^. An electrostatic filter was installed in front of each instrument to remove ions and charged clusters formed in the chamber. Sulfuric acid and HOMs were quantified following calibration and loss correction procedures described previously^19,22,39^.

VOCs were monitored by a proton transfer reaction time-of-flight mass spectrometer (PTR-TOF-MS; Ionicon Analytik); this also provides information about the overall cleanliness regarding VOCs in the chamber. The technique has been extensively described previously^40^. Direct calibration using diffusion sources allows us to determine VOC mixing ratios with an accuracy of 5% and a typical detection limit of 25 pptv (ref. ^41^).

Gas monitors were used to measure ozone (O_3_; Thermo Environmental Instruments TEI 49C), sulfur dioxide (SO_2_; Thermo Fisher Scientific 42i-TLE) and nitric oxide (NO; ECO Physics, CLD 780TR). Nitrogen dioxide (NO_2_) was measured using a cavity-attenuated phase-shift NO_2_ monitor (CAPS NO_2_, Aerodyne Research) and a homemade cavity-enhanced differential optical absorption spectroscopy (CE-DOAS) instrument. The relative humidity of the chamber was determined using dew point mirrors (EdgeTech).

We measured the particle-phase composition via thermal desorption using an iodide-adduct chemical ionization time-of-flight mass spectrometer equipped with a filter inlet for gases and aerosols (FIGAERO-CIMS)^42,43^. FIGAERO is a manifold inlet for a CIMS with two operating modes. In one mode, gases are directly sampled into a 100-mbar turbulent flow ion-molecule reactor while particles are concurrently collected on a polytetrafluorethylene (PTFE) filter via a separate dedicated port. In the other mode, the filter is automatically moved into a pure N_2_ gas stream flowing into the ion-molecule reactor while the N_2_ is progressively heated to evaporate the particles via temperature-programmed desorption. Analytes are then chemically ionized and extracted into a TOF-MS, achieving a detection limit below 10^6^ cm^−3^.

Particle-size distributions between 1.8 nm and 500 nm were monitored continuously by a differential mobility analyser train (DMA-Train), a nano-scanning electrical mobility spectrometer (nSEMS), a nano-scanning mobility particle sizer (nano-SMPS), and a long-SMPS. The DMA-Train was constructed with six identical DMAs operating at different, but fixed, voltages. Particles transmitted through the DMAs were then detected by either a particle-size magnifier (PSM) or a CPC, depending on the size ranges. An approximation of the size distribution with 15 size bins was acquired by logarithmic interpolation between the six channels^44^. The nSEMS used a new, radial opposed migration ion and aerosol classifier (ROMIAC), which is less sensitive to diffusional resolution degradation than the DMAs^45^, and a soft X-ray charge conditioner. After leaving the classifier, particles were first activated in a fast-mixing diethylene glycol stage^46^, and then counted with a butanol-CPC. The nSEMS transfer function that was used to invert the data to obtain the particle-size distribution was derived using three-dimensional finite-element modelling of the flows, electric field and particle trajectories^47,48^. The two commercial mobility particle-size spectrometers, nano-SMPS and long-SMPS, have been fully characterized, calibrated and validated in several previous studies^49–51^.

### Determination of growth rate

**Extended Data Fig. 5:**
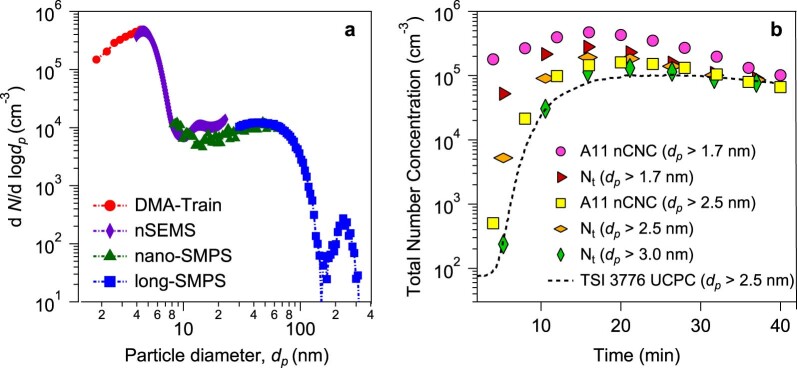
Combined particle-size distribution and total concentrations from four particle characterization instruments. **a**, Combined size distributions, ${n}_{N}^{^\circ }({d}_{{\rm{p}}})={\rm{d}}N/{\rm{d}}(\log {d}_{{\rm{p}}})$, from four electrical mobility particle-size spectrometers of different, but overlapping, detection ranges. The DMA-Train, nSEMS and nano-SMPS data were averaged every five minutes to coordinate with the long-SMPS scanning time resolution. The tail of the size distribution of large particles outside the detection range was extrapolated by fitting a lognormal distribution. **b**, Comparison of the integrated number concentrations from the combined size distributions in **a** with total number counts obtained from fixed-cutoff-size condensation particle counters. We obtained the total number concentration of particles, *N*_t_(*d*_p0_), above a cutoff size, *d*_p0_, by integrating the particle-size distribution using^52^: ${N}_{t}={\int }_{{d}_{{p}_{0}}}^{{\rm{\infty }}}\{{n}_{N}({d}_{{\rm{p}}})\times {\eta }_{{\rm{U}}{\rm{C}}{\rm{P}}{\rm{C}}}\}{\rm{d}}{d}_{{\rm{p}}}$, applying the size-dependent detection efficiency, *η*_UCPC_^61^, to adjust the integrated total number concentration. We plot the total number concentrations for three different cutoff sizes (*d*_*p*0_) of 1.7 nm, 2.5 nm and 3.0 nm, obtained every 5 min, with coloured symbols as shown in the legend. We also plot measured total number concentrations from two instruments: the Airmodus A11 nCNC-system at nominal cutoff sizes of 1.7 nm and 2.5 nm, and a TSI 3776 UCPC with a nominal cutoff size of 2.5 nm. The Airmodus A11 nCNC-system consists of an A10 PSM and an A20 CPC, which determined both the size distribution of 1–4-nm aerosol particles and the total number concentration of particles smaller than 1 μm (ref. ^62^). The TSI 3776 UCPC has a rapid response time and so, rather than the 5-min basis for the other points, we plot the values from this instrument with a dashed curve. Source Data

**Extended Data Fig. 6:**
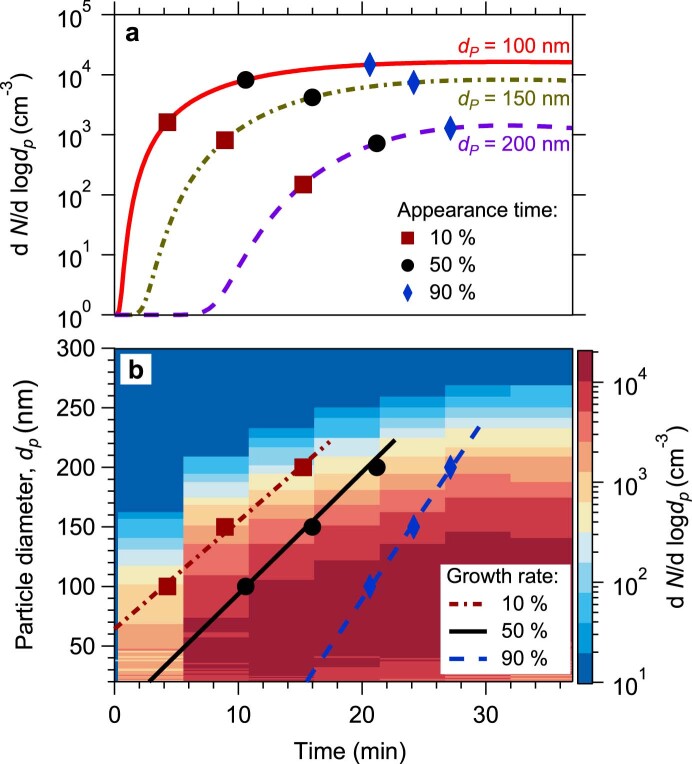
Determination of growth rate using the appearance-time method. **a**, Logarithmic interpolated time-dependent growth profiles for particles with diameters of 100 nm, 150 nm and 200 nm. Three appearance times, when particle number concentrations reached 10%, 50%, and 90% of their maximum, are labelled with different symbols for the three different diameters. **b**, Growth-rate calculation for a rapid growth event (as in Fig. 2) above the activation diameter. The growth rates, in nm h^−1^, that we report here are the slopes of linear fits to the 50% (among 10%, 50% and 90%) appearance times calculated from all sizes above the activation diameter (the slope of the solid black line and the black circles in **b**). Source Data

The combined particle-size distribution was reconstructed using measurement data from DMA-Train at 1.8–4.3 nm, nSEMS at 4.3–18.1 nm, nano-SMPS at 18.1–55.2 nm and long-SMPS at 55.2–500 nm, and synchronized with long-SMPS measurement time. We list the sizing and resolution information of these instruments in Extended Data Table 2. As depicted in Extended Data Fig. 5a, the four instruments showed excellent agreement in their overlapping regions of the size ranges. The total number concentration obtained by integrating the combined size distribution agreed well with measurement by an Airmodus A11 nano-condensation nucleus counter (nCNC) and a TSI 3776 ultrafine condensation particle counter (UCPC) (Extended Data Fig. 5b). Particle growth rate, d*d*_p_/d*t*, was then determined from the combined size distributions using the 50% appearance time method^20^, as a clear front of a growing particle population could be identified during most rapid growth events (Extended Data Fig. 6). For the rapid growth rates, which are the principal focus here, the SMPS measurements provided the major constraint.

### Determination of activation diameter

The activation diameter (*d*_act_) was interpreted as the size at which growth accelerated from the slow, initial rate to the rapid, post-activation rate. The activation diameter was determined using the particle-size distribution acquired from DMA-Train or nSEMS at small sizes (less than 15 nm). At the activation diameter, the growth rate calculated from the 50% appearance time usually experienced a sharp change, from below 10 nm h^−1^ to (often) over 100 nm h^−1^, depending on concentrations of supersaturated HNO_3_ and NH_3_ vapours. A fast growth rate leads to a relatively low steady-state concentration of particles just above the activation diameter; the activation event often resulted in a notable gap in the particle-number size distribution. In some cases, a clear bimodal distribution was observed, with the number concentration in one size bin plunging below 10 counts cm^−3^, while the counts at larger sizes rose to more than 100 counts cm^−3^; the centroid diameter of the size bin at which the number concentration dropped was then defined as the activation diameter (Extended Data Fig. 2a).

### Calculation of saturation ratio

We model the ammonium nitrate formed in the particle phase as solid in our particle growth experiments, given that the relative humidity (roughly 60%) was less than the deliquescence relative humidity (DRH), given by^52^:

$$\mathrm{ln}(\text{DRH})=\frac{723.7}{T}+1.6954$$

The dissociation constant, *K*_p_, is defined as the product of the equilibrium partial pressures of HNO_3_ and NH_3_. *K*_p_ can be estimated by integrating the van’t Hoff equation^53^. The resulting equation for *K*_p_ in units of ppb^2^ (assuming 1 atm of total pressure)^54^ is:

$${\rm{l}}{\rm{n}}{K}_{{\rm{p}}}=118.87-\frac{\mathrm{24,084}}{T}-6.025\,{\rm{l}}{\rm{n}}T$$

**Extended Data Fig. 7:**
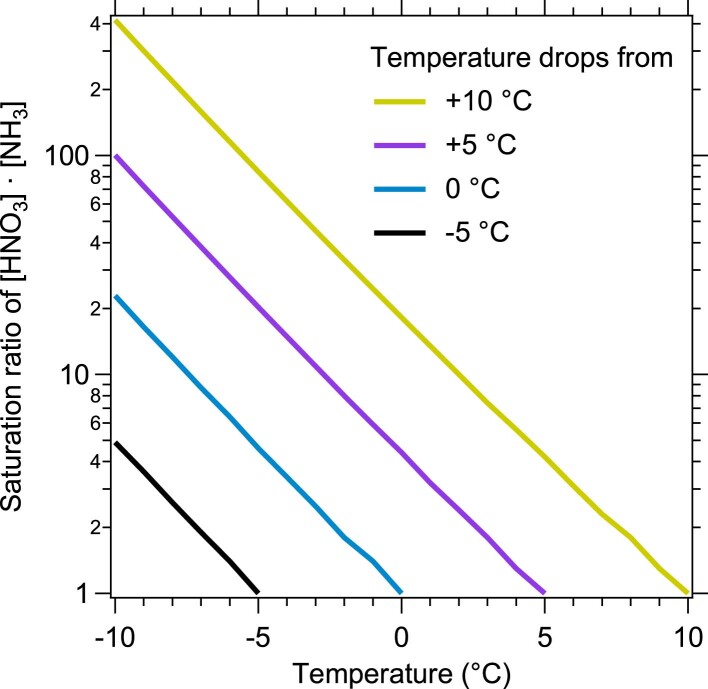
Saturation ratio as a function of temperature. At constant nitric acid and ammonia, a decline in temperature leads to an exponential increase in the saturation ratio of ammonium nitrate, shown as the product of nitric acid and ammonia vapour concentration. With an adiabatic lapse rate of −9 °C km^−1^ during adiabatic vertical mixing, upward transport of a few hundred metres alone is enough for a saturated nitric acid and ammonia air parcel to reach the saturation ratio capable of triggering rapid growth at a few nanometres. Source Data

The saturation ratio, *S*, is thus calculated by dividing the product of measured mixing ratios of HNO_3_ and NH_3_ by the dissociation constant. The dissociation constant is quite sensitive to temperature changes, varying over more than two orders of magnitude for typical ambient conditions. Several degrees of temperature drop can lead to a much higher saturation ratio, shifting the equilibrium of the system towards the particle phase drastically. As illustrated in Extended Data Fig. 7, with an adiabatic lapse rate of −9 °C km^−1^ during fast vertical mixing, upward transport of a few hundred metres alone is sufficient for a saturated nitric acid and ammonia air parcel to reach the saturation ratio capable of triggering rapid growth at a few nanometres.

### Determination of nucleation rate

The nucleation rate, *J*_1.7_, is determined here at a mobility diameter of 1.7 nm (a physical diameter of 1.4 nm) using particle size magnifier (PSM). At 1.7 nm, a particle is normally considered to be above its critical size and, therefore, thermodynamically stable. *J*_1.7_ is calculated using the flux of the total concentration of particles growing past a specific diameter (here at 1.7 nm), as well as correction terms accounting for aerosol losses due to dilution in the chamber, wall losses and coagulation. Details can be found in our previous work^55^.

### The MABNAG model

To compare our measurements to thermodynamic predictions (including the Kelvin term for curved surfaces), we used the model for acid-base chemistry in nanoparticle growth (MABNAG)^25^. MABNAG is a monodisperse particle population growth model that calculates the time evolution of particle composition and size on the basis of concentrations of condensing gases, relative humidity and ambient temperature, considering also the dissociation and protonation between acids and bases in the particle phase. In the model, water and bases are assumed always to be in equilibrium state between the gas and particle phases. Mass fluxes of acids to and from the particles are determined on the basis of their gas phase concentrations and their equilibrium vapour concentrations. In order to solve for the dissociation- and composition-dependent equilibrium concentrations, MABNAG couples a particle growth model to the extended aerosol inorganics model (E-AIM)^56,57^. Here, we assumed particles in MABNAG to be liquid droplets at +5 °C and −10 °C, at 60% relative humidity. The simulation system consisted of four compounds: water, ammonia, sulfuric acid and nitric acid. The initial particle composition in each simulation was 40 sulfuric acid molecules and a corresponding amount of water and ammonia according to gas-particle equilibrium on the basis of their gas concentrations. With this setting, the initial diameter was approximately 2 nm. Particle density and surface tension were set to 1,500 kg m^−3^ and 0.03 N m^−1^, respectively. In Extended Data Fig. 4, we show that MAGNAG computations confirm that nitric acid and ammonia at the measured concentrations can activate small particles and cause rapid growth, and also confirm that the activation diameter depends on the ammonium nitrate saturation ratio, consistent with our measured diameter (diamonds in Fig. 3a).

### Nano-Köhler theory

To prove consistency, we also calculated the equilibrium saturation ratios of ammonium nitrate above curved particle surfaces according to nano-Köhler theory^23^. This theory describes the activation of nanometre-sized inorganic clusters to growth by vapour condensation, which is analogous to Köhler theory describing the activation of cloud condensation nuclei (CCN) to cloud droplets. Here, we assumed seed particles of ammonium sulfate, and performed calculations for three seed-particle diameters (*d*_s_ = 1.4 nm, 2.0 nm and 2.9 nm) at +5 °C and −10 °C, and at 60% relative humidity. The equilibrium vapour pressures of HNO_3_ and NH_3_ over the liquid phase, and the surface tension and density of the liquid phase, were obtained from an E-AIM^56,57^. The equilibrium saturation ratios of ammonium nitrate were calculated as described in the Methods section ‘Calculation of saturation ratio’, also including the Kelvin term. The resulting Köhler curves, showing the equilibrium saturation ratio as a function of particle diameter, are presented in Extended Data Fig. 2c. The maxima of each curve corresponds to the activation diameter (*d*_act_); saturation ratios of 10–50 lead to *d*_act_ values of 3–5 nm, consistent with our measurements in Fig. 3a. We summarize detailed results in Extended Data Table 1.

### Ambient nucleation and growth

In Extended Data Table 3 we compile ambient observations of nucleation rates, growth rates and the ambient condensation sink. In most cases these are derived from evolving particle-size distributions. We summarize these observations in Extended Data Fig. 1.

## Online content

Any methods, additional references, Nature Research reporting summaries, source data, extended data, supplementary information, acknowledgements, peer review information; details of author contributions and competing interests; and statements of data and code availability are available at 10.1038/s41586-020-2270-4.

## Source data

Source Data Fig. 1

Source Data Fig. 2

Source Data Fig. 3

Source Data Fig. 4

Source Data Extended Data Fig. 1

Source Data Extended Data Fig. 2

Source Data Extended Data Fig. 3

Source Data Extended Data Fig. 4

Source Data Extended Data Fig. 5

Source Data Extended Data Fig. 6

Source Data Extended Data Fig. 7

## Figures and Tables

**Extended Data Table 1:**
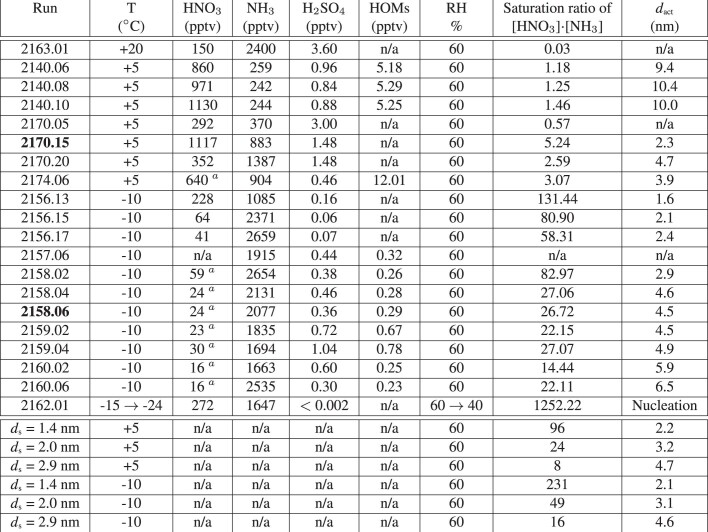
Conditions for all nucleation and growth experiments and nano-Köhler simulations discussed here

^a^HNO_3_ production via NO_2_ photo-oxidation.

n/a, not applicable; RH, relative humidity.

**Extended Data Table 2:**
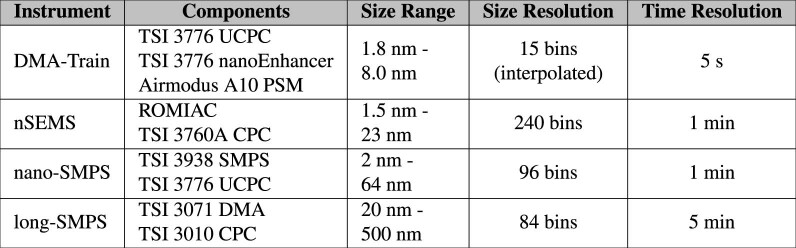
Specifications of the four particle-sizing instruments used here

**Extended Data Table 3:**
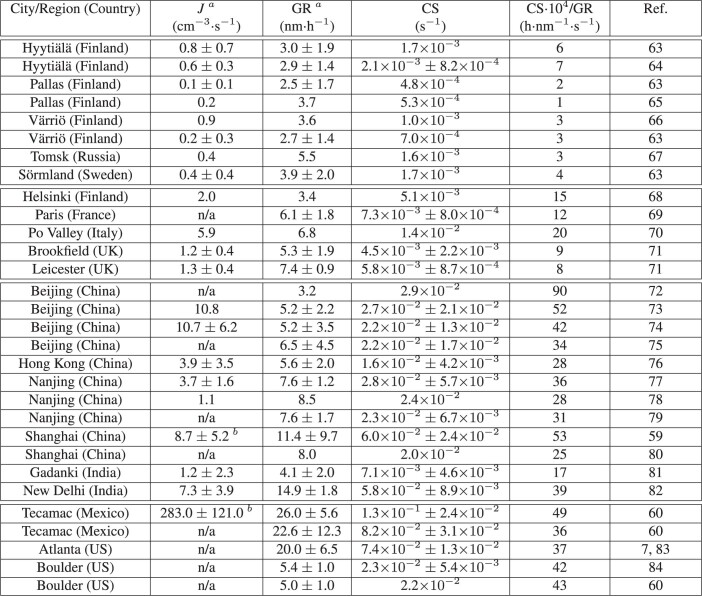
Ambient particle-formation rates (*J*), growth rates (GR) and condensation sinks (CS) in various remote and urban environments

^a^*J* and GR were mostly calculated over a size range from a few nanometres to more than 20 nm.

^b^*J* calculated from 3 nm to 6 nm.

Uncertainties indicate 1*σ* errors. From refs. ^7,59,60,63–84^.

## Data Availability

The full dataset shown in the figures and tables is publicly available^58^. All data shown in the figures and tables and additional raw data are available upon request from the corresponding author. Source data for Figs. 1–4 and Extended Data Figs. 1–7 are provided with the paper.

## Code availability

Codes for the MABNAG and nano-Köhler simulations and for conducting the analysis presented here can be obtained upon request from the corresponding author.

## References

[1] Stanier, C. O., Khlystov, A. Y. & Pandis, S. N. Nucleation events during the Pittsburgh Air Quality Study: description and relation to key meteorological, gas phase, and aerosol parameters. *Aerosol Sci. Technol*. **38**, 253–264 (2004).

[2] Yao, L. et al. Atmospheric new particle formation from sulfuric acid and amines in a Chinese megacity. *Science* **361**, 278–281 (2018). 30026225 10.1126/science.aao4839

[3] Kulmala, M., Kerminen, V.-M., Petäjä, T., Ding, A. J. & Wang, L. Atmospheric gas-to-particle conversion: why NPF events are observed in megacities? *Faraday Discuss*. **200**, 271–288 (2017). 28573268 10.1039/c6fd00257a

[4] Höpfner, M. et al. Ammonium nitrate particles formed in upper troposphere from ground ammonia sources during Asian monsoons. *Nat. Geosci*. **12**, 608–612 (2019).

[5] Williamson, C. J. et al. A large source of cloud condensation nuclei from new particle formation in the tropics. *Nature* **574**, 399–403 (2019). 31619794 10.1038/s41586-019-1638-9

[6] Intergovernmental Panel on Climate Change (IPCC). *Climate Change 2013: The Physical Science Basis* (Cambridge Univ. Press, 2013).

[7] McMurry, P. H. et al. A criterion for new particle formation in the sulfur-rich Atlanta atmosphere. *J. Geophys. Res. D* **110**, D22S02 (2005).

[8] Kulmala, M. et al. Direct observations of atmospheric aerosol nucleation. *Science* **339**, 943–946 (2013). 23430652 10.1126/science.1227385

[9] Gordon, H. et al. Causes and importance of new particle formation in the present-day and pre-industrial atmospheres. *J. Geophys. Res. D* **122**, 8739–8760 (2017).

[10] Riipinen, I. et al. Contribution of organics to atmospheric nanoparticle growth. *Nat. Geosci*. **5**, 453–458 (2012).

[11] Pierce, J. R. & Adams, P. J. Efficiency of cloud condensation nuclei formation from ultrafine particles. *Atmos. Chem. Phys*. **7**, 1367–1379 (2007).

[12] Kuang, C., McMurry, P. H. & McCormick, A. V. Determination of cloud condensation nuclei production from measured new particle formation events. *Geophys. Res. Lett*. **36**, L09822 (2009).

[13] Apte, J. S., Brauer, M., Cohen, A. J., Ezzati, M. & Pope, C. A. Ambient PM_2.5_ reduces global and regional life expectancy. *Environ. Sci. Technol. Lett*. **5**, 546–551 (2018).

[14] Chen, G., Wang, W.-C. & Chen, J.-P. Circulation responses to regional aerosol climate forcing in summer over East Asia. *Clim. Dyn*. **51**, 3973–3984 (2018).

[15] Kerminen, V.-M. & Kulmala, M. Analytical formulae connecting the “real” and the “apparent” nucleation rate and the nuclei number concentration for atmospheric nucleation events. *J. Aerosol Sci*. **33**, 609–622 (2002).

[16] Takahama, S., Wittig, A. E., Vayenas, D. V., Davidson, C. I. & Pandis, S. N. Modeling the diurnal variation of nitrate during the Pittsburgh Air Quality Study. *J. Geophys. Res. D* **109**, D16S06 (2004).

[17] Xu, W. et al. Changes in aerosol chemistry from 2014 to 2016 in winter in Beijing: insights from high-resolution aerosol mass spectrometry. *J. Geophys. Res. D* **124**, 1132–1147 (2019).

[18] McMurry, P. H. Photochemical aerosol formation from SO_2_: a theoretical analysis of smog chamber data. *J. Colloid Interface Sci*. **78**, 513–527 (1980).

[19] Kirkby, J. et al. Ion-induced nucleation of pure biogenic particles. *Nature* **533**, 521–526 (2016). 27225125 10.1038/nature17953 PMC8384037

[20] Stolzenburg, D. et al. Rapid growth of organic aerosol nanoparticles over a wide tropospheric temperature range. *Proc. Natl Acad. Sci. USA* **115**, 9122–9127 (2018). 30154167 10.1073/pnas.1807604115 PMC6140529

[21] O’Dowd, C. D. et al. Marine aerosol formation from biogenic iodine emissions. *Nature* **417**, 632–636 (2002). 12050661 10.1038/nature00775

[22] Kirkby, J. et al. Role of sulphuric acid, ammonia and galactic cosmic rays in atmospheric aerosol nucleation. *Nature* **476**, 429–433 (2011). 21866156 10.1038/nature10343

[23] Kontkanen, J., Olenius, T., Kulmala, M. & Riipinen, I. Exploring the potential of nano-köhler theory to describe the growth of atmospheric molecular clusters by organic vapors using cluster kinetics simulations. *Atmos. Chem. Phys*. **18**, 13733–13754 (2018).

[24] Lu, K. et al. Fast photochemistry in wintertime haze: consequences for pollution mitigation strategies. *Environ. Sci. Technol*. **53**, 10676–10684 (2019). 31418557 10.1021/acs.est.9b02422

[25] Yli-Juuti, T. et al. Model for acid-base chemistry in nanoparticle growth (MABNAG). *Atmos. Chem. Phys*. **13**, 12507–12524 (2013).

[26] Letzel, M. O. et al. LES case study on pedestrian level ventilation in two neighbourhoods in Hong Kong. *Meteorol. Z. (Berl.)* **21**, 575–589 (2012).

[27] Manninen, H. E. et al. EUCAARI ion spectrometer measurements at 12 European sites – analysis of new particle formation events. *Atmos. Chem. Phys*. **10**, 7907–7927 (2010).

[28] Pierce, J. R. et al. Quantification of the volatility of secondary organic compounds in ultrafine particles during nucleation events. *Atmos. Chem. Phys*. **11**, 9019–9036 (2011).

[29] Ge, C., Zhu, C., Francisco, J. S., Zeng, X. C. & Wang, J. A molecular perspective for global modeling of upper atmospheric NH_3_ from freezing clouds. *Proc. Natl Acad. Sci. USA* **115**, 6147–6152 (2018). 29848636 10.1073/pnas.1719949115 PMC6004466

[30] Liu, L. et al. The role of nitric acid in atmospheric new particle formation. *Phys. Chem. Chem. Phys*. **20**, 17406–17414 (2018). 29911231 10.1039/c8cp02719f

[31] Duplissy, J. et al. Effect of ions on sulfuric acid-water binary particle formation: 2. Experimental data and comparison with QC-normalized classical nucleation theory. *J. Geophys. Res. D* **121**, 1752–1775 (2016).

[32] Dias, A. et al. Temperature uniformity in the CERN CLOUD chamber. Aerosol *Meas. Tech*. **10**, 5075–5088 (2017).

[33] Schnitzhofer, R. et al. Characterisation of organic contaminants in the CLOUD chamber at CERN. *Aerosol Meas. Techn*. **7**, 2159–2168 (2014).

[34] Jokinen, T. et al. Atmospheric sulphuric acid and neutral cluster measurements using CI-APi-TOF. *Atmos. Chem. Phys*. **12**, 4117–4125 (2012).

[35] Junninen, H. et al. A high-resolution mass spectrometer to measure atmospheric ion composition. *Atmos. Meas. Tech*. **3**, 1039–1053 (2010).

[36] Eisele, F. & Tanner, D. Measurement of the gas phase concentration of H_2_SO_4_ and methane sulfonic acid and estimates of H_2_SO_4_ production and loss in the atmosphere. *J. Geophys. Res. D* **98**, 9001–9010 (1993).

[37] Pfeifer, J. et al. Measurement of ammonia, amines and iodine species using protonated water cluster chemical ionization mass spectrometry. *Atmos. Meas. Tech*. 10.5194/amt-2019-215 (2019).

[38] Kürten, A., Rondo, L., Ehrhart, S. & Curtius, J. Performance of a corona ion source for measurement of sulfuric acid by chemical ionization mass spectrometry. *Atmos. Meas. Tech*. **4**, 437–443 (2011).

[39] Tröstl, J. et al. The role of low-volatility organic compounds in initial particle growth in the atmosphere. *Nature* **533**, 527–531 (2016). 27225126 10.1038/nature18271 PMC8384036

[40] Breitenlechner, M. et al. PTR3: an instrument for studying the lifecycle of reactive organic carbon in the atmosphere. *Anal. Chem*. **89**, 5824–5831 (2017). 28436218 10.1021/acs.analchem.6b05110

[41] Gautrois, M. & Koppmann, R. Diffusion technique for the production of gas standards for atmospheric measurements. *J. Chromatogr. A* **848**, 239–249 (1999).

[42] Wang, M. et al. Reactions of atmospheric particulate stabilized Criegee intermediates lead to high molecular weight aerosol components. *Environ. Sci. Technol*. **50**, 5702–5710 (2016). 27186797 10.1021/acs.est.6b02114

[43] Lopez-Hilfiker, F. D. et al. A novel method for online analysis of gas and particle composition: description and evaluation of a Filter Inlet for Gases and AEROsols (FIGAERO). *Atmos. Meas. Tech*. **7**, 983–1001 (2014).

[44] Stolzenburg, D., Steiner, G. & Winkler, P. M. A DMA-train for precision measurement of sub-10 nm aerosol dynamics. *Atmos. Meas. Tech*. **10**, 1639–1651 (2017).

[45] Mui, W., Mai, H., Downard, A. J., Seinfeld, J. H. & Flagan, R. C. Design, simulation, and characterization of a radial opposed migration ion and aerosol classifier (ROMIAC). *Aerosol Sci. Technol*. **51**, 801–823 (2017).

[46] Wimmer, D. et al. Performance of diethylene glycol-based particle counters in the sub-3 nm size range. *Atmos. Meas. Tech*. **6**, 1793–1804 (2013).

[47] Mai, H. & Flagan, R. C. Scanning DMA data analysis I. Classification transfer function. *Aerosol Sci. Technol*. **52**, 1382–1399 (2018).

[48] Mai, H., Kong, W., Seinfeld, J. H. & Flagan, R. C. Scanning DMA data analysis II. Integrated DMA-CPC instrument response and data inversion. *Aerosol Sci. Technol*. **52**, 1400–1414 (2018).

[49] Jurányi, Z. et al. A 17 month climatology of the cloud condensation nuclei number concentration at the high alpine site Jungfraujoch. *J. Geophys. Res. D* **116**, D10204 (2011).

[50] Tröstl, J. et al. Fast and precise measurement in the sub-20 nm size range using a scanning mobility particle sizer. *J. Aerosol Sci*. **87**, 75–87 (2015).

[51] Wiedensohler, A. et al. Mobility particle size spectrometers: harmonization of technical standards and data structure to facilitate high quality long-term observations of atmospheric particle number size distributions. *Atmos. Meas. Tech*. **5**, 657–685 (2012).

[52] Seinfeld, J. H. & Pandis, S. N. *Atmospheric Chemistry and Physics* 2nd edn (John Wiley & Sons, 2006).

[53] Denbigh, K. G. & Denbigh, K. G. *The Principles of Chemical Equilibrium: With Applications in Chemistry and Chemical Eengineering* (Cambridge Univ. Press, 1981).

[54] Mozurkewich, M. The dissociation constant of ammonium nitrate and its dependence on temperature, relative humidity and particle size. *Atmos. Environ. A* **27**, 261–270 (1993).

[55] Lehtipalo, K. et al. Multi-component new particle formation from sulfuric acid, ammonia and biogenic vapors. *Sci. Adv*. **4**, eaau5363 (2018). 30547087 10.1126/sciadv.aau5363 PMC6291317

[56] Clegg, S. L. & Seinfeld, J. H. Thermodynamic models of aqueous solutions containing inorganic electrolytes and dicarboxylic acids at 298.15 K. 1. The acids as nondissociating components. *J. Phys. Chem. A* **110**, 5692–5717 (2006). 16640364 10.1021/jp056149k

[57] Clegg, S. L. & Seinfeld, J. H. Thermodynamic models of aqueous solutions containing inorganic electrolytes and dicarboxylic acids at 298.15 K. 2. Systems including dissociation equilibria. *J. Phys. Chem. A* **110**, 5718–5734 (2006). 16640365 10.1021/jp056150j

[58] Wang, M. et al. Rapid growth of new atmospheric particles by nitric acid and ammonia condensation: data resources. 10.5281/zenodo.3653377 (2020). 10.1038/s41586-020-2270-4 PMC7334196 32405020

[59] Xiao, S. et al. Strong atmospheric new particle formation in winter in urban Shanghai, China. *Atmos. Chem. Phys*. **15**, 1769–1781 (2015).

[60] Iida, K., Stolzenburg, M. R., McMurry, P. H. & Smith, J. N. Estimating nanoparticle growth rates from size-dependent charged fractions: Analysis of new particle formation events in Mexico City. *J. Geophys. Res. D Atmospheres* **113**, D05207 (2008).

[61] Mordas, G. et al. On operation of the ultra-fine water-based CPC TSI 3786 and comparison with other TSI models (TSI 3776, TSI 3772, TSI 3025, TSI 3010, TSI 3007). *Aerosol Sci. Technol*. **42**, 152–158 (2008).

[62] Lehtipalo, K. et al. The effect of acid-base clustering and ions on the growth of atmospheric nano-particles. *Nat. Commun*. **7**, 11594 (2016). 27197574 10.1038/ncomms11594 PMC4876472

[63] Dal Maso, M. et al. Aerosol size distribution measurements at four Nordic field stations: identification, analysis and trajectory analysis of new particle formation bursts. *Tellus B Chem. Phys. Meterol*. **59**, 350–361 (2007).

[64] Dal Maso, M. et al. Formation and growth of fresh atmospheric aerosols: eight years of aerosol size distribution data from SMEAR II, Hyytiälä, Finland. *Boreal Environ. Res*. **10**, 323–326 (2005).

[65] Komppula, M. et al. Observations of new particle formation and size distributions at two different heights and surroundings in subarctic area in northern finland. *J. Geophys. Res. D Atmospheres* **108** (D9), 4295 (2003).

[66] Vehkamäki, H. et al. Atmospheric particle formation events at Värriö measurement station in Finnish Lapland 1998-2002. *Atmos. Chem. Phys*. **4**, 2015–2023 (2004).

[67] Dal Maso, M. et al. Aerosol particle formation events at two Siberian stations inside the boreal forest. *Boreal Environ. Res*. **13**, 81–92 (2008).

[68] Hussein, T. et al. Observation of regional new particle formation in the urban atmosphere. *Tellus B Chem. Phys. Meterol*. **60**, 509–521 (2008).

[69] Pikridas, M. et al. In situ formation and spatial variability of particle number concentration in a European megacity. *Atmos. Chem. Phys*. **15**, 10219–10237 (2015).

[70] Hamed, A. et al. Nucleation and growth of new particles in Po Valley, Italy. *Atmos. Chem. Phys*. **7**, 355–376 (2007).

[71] Hama, S. M., Cordell, R. L., Kos, G. P., Weijers, E. & Monks, P. S. Sub-micron particle number size distribution characteristics at two urban locations in leicester. *Atmos. Res*. **194**, 1–16 (2017).

[72] Gao, J., Chai, F., Wang, T., Wang, S. & Wang, W. Particle number size distribution and new particle formation: new characteristics during the special pollution control period in Beijing. *J. Environ. Sci*. **24**, 14–21 (2012). 10.1016/s1001-0742(11)60725-0 22783611

[73] Wang, Z. et al. Characteristics of regional new particle formation in urban and regional background environments in the North China Plain. *Atmos. Chem. Phys*. **13**, 12495–12506 (2013).

[74] Yue, D. et al. Characteristics of aerosol size distributions and new particle formation in the summer in Beijing. *J. Geophys. Res. D Atmospheres* **114**, D00G12 (2009).

[75] Zhang, Y. et al. Characterization of new particle and secondary aerosol formation during summertime in Beijing, *China. Tellus B Chem. Phys. Meterol*. **63**, 382–394 (2011).

[76] Man, H. et al. Comparison of daytime and nighttime new particle growth at the HKUST supersite in Hong Kong. *Environ. Sci. Technol*. **49**, 7170–7178 (2015). 25988913 10.1021/acs.est.5b02143

[77] An, J. et al. Characteristics of new particle formation events in Nanjing, China: effect of water-soluble ions. *Atmos. Environ*. **108**, 32–40 (2015).

[78] Herrmann, E. et al. Aerosols and nucleation in eastern China: first insights from the new SORPES-NJU station. *Atmos. Chem. Phys*. **14**, 2169–2183 (2014).

[79] Yu, H. et al. Nucleation and growth of sub-3 nm particles in the polluted urban atmosphere of a megacity in China. *Atmos. Chem. Phys*. **16**, 2641–2657 (2016).

[80] Peng, J. et al. Submicron aerosols at thirteen diversified sites in China: size distribution, new particle formation and corresponding contribution to cloud condensation nuclei production. *Atmos. Chem. Phys*. **14**, 10249–10265 (2014).

[81] Kanawade, V. et al. Infrequent occurrence of new particle formation at a semi-rural location, Gadanki, in tropical Southern India. *Atmos. Environ*. **94**, 264–273 (2014).

[82] Mönkkönen, P. et al. Measurements in a highly polluted Asian mega city: observations of aerosol number size distribution, modal parameters and nucleation events. *Atmos. Chem. Phys*. **5**, 57–66 (2005).

[83] Kuang, C. et al. An improved criterion for new particle formation in diverse atmospheric environments. *Atmos. Chem. Phys*. **10**, 8469–8480 (2010).

[84] Iida, K. et al. Contribution of ion-induced nucleation to new particle formation: Methodology and its application to atmospheric observations in Boulder, Colorado. *J. Geophys. Res. D Atmospheres* **111**, D23201 (2006).

